# Modern Sociology

**Published:** 1899-07-08

**Authors:** 


### Modern Sociology.

### PEOPLE'S BANK IN IRELAND.

In speaking some time ago about people's banks, and the great success they have had on the Continent, we expressed a doubt if such institutions were suitable for Britain. But we forgot that there is one part of the kingdom where the conditions are very similar to those of the continental countries where these banks have proved such a boon. We mean Ireland. Ireland is a country of small farmers, who have but little capital, and who, partly on that account and partly from ignorance, follow antiquated methods. Now the only way in which the small man can compete with the big man possessed of large capital in any industry is by co-operation. A score of poor men, putting their "pickles" together, can make a "mickle" equal to that of the rich man. The scanty hoardings of a hundred old stockings or teapots may, if put together, bring more money to those who hoarded, and at the same time open up the road to fortune to a more energetic and capable member of the community. This is, in brief, the whole history of banking, the only weak point of which is that it has hardly ever been put into practice in this country on a sufficiently microscopic scale. But Mr. Horace Plunkett has now introduced the village bank into Ireland, and with such success that even Nationalists, who, politically, differ from him on many points, are ready to acknowledge the beneficence of his work. It is only after ten years of hard work that Mr. Plunkett has succeeded in making an impression on the traditional languor and fatalism of Ireland. He addressed fifty meetings before he managed to found even one little society to carry out his ideas. But the result of his perseverance is found to-day in the fact that there are 340 agricultural societies, with a membership of 40,000 farmers. These societies, with their united capital, have money to buy the machinery by means of which alone agriculture can be profitably carried on in face of the competition of other countries. Sending a large amount of produce to one market, these societies can get cheaper railway rates than each member of them could do privately. Co-operation, by rousing farmers from the dull isolation into which they had fallen, makes them more ready to study new methods and make experiments in farming. And, not least, it has taught them to put their capital together, to lend it out to men whom they know, and who will put it to a productive use. Mr. Plunkett's banks are, for the most part, parish banks. Thus the persons who want to borrow money are known personally to the committee, while, wherever it is practicable, the committee is composed of men who are not likely to require to borrow. Thus a man does not get on to the committee of the parish bank to serve his own ends. The universal rule is that the money must be borrowed for a specific object, to which it must be devoted. That object must be productive; and, by limiting the radius of the bank's operations to so small a field as the parish, it is possible to see that the borrower fulfils the conditions of the loan. In one little bank in the poorest part of County Mayo, the village of Belmullet, the total capital of the bank amounts to £235. But it is able to give 4 per cent. to depositors, lends money at 5 per cent., and has never had a single bad debt. The borrowers are full of its praises. One man borrowed £2 from it, and made a profit of £7 in five months; another got £6, on which his gross earnings were £20, and so on. These banks, wherever they have been established, have raised men who were on the verge of bankruptcy to comparative wealth, and maintained their independence. More than that, they are teaching the Irish that, though political agitation may have its uses, it will not of itself till their land nor fill their pockets. The banks are teaching those principles of industry, self-help, and forethought by which alone it will come about that Ireland will cease to be a "distressful country."

---

### OLD AGE PENSION RECOMMENDATIONS.

The question of Old Age Pensions is a hard nut to crack. The recommendation that no one should be eligible for a pension who has during the twenty years previous to the application for a pension been a recipient of poor-law relief, is sound, though the concession that this restriction may be ignored under "exceptional circumstances" is almost certain to lead to a variety of treatment in identical cases, according to the temper of the local authorities who decide what constitutes "exceptional circumstances." Moreover, women, especially widows with children, even though they are respectable and industrious, are often compelled to accept poor-law relief, and the recommendation as a whole will not meet the views of those who by old age pensions mean only poor-law relief, without the accompanying disqualifications, which at present more or less prevent that kind of pension from abuse. The recommendation which disqualifies for a pension anyone who has been imprisoned without the option of a fine within the same period wisely excludes the criminal class, while it does not permit the raking up against a man those indiscretions of youth which may lead a man into the police court as much through some ebullition of high spirits as through *malice prepense*. But even in these cases of more recent imprisonment, "exceptional circumstances" may permit of an application being favourably considered. But we must admit that we think the age at which the pensions begin is too high. Very few of the labouring class are able to maintain themselves wholly after "the eye is dim, and the natural strength abated," as it admittedly is at the age of sixty-five. Intellectual power survives muscular energy, yet sixty-five is the age when the members of the various Government services are compelled to retire; and the lower a man's work lies in the intellectual scale, the more dependent it is on mere physical strength, the earlier comes the age for superannuation. Surely, if the scheme is to benefit the poorest class, the age at which the pension should commence should not be more than sixty. Especially if the clause be adopted which permits the giving of a pension to one in receipt of wages not exceeding five shillings a week, does it seem reasonable that something under sixty-five should be the minimum age. The man who is earning five shillings a week at sixty-five will not have been earning much more in the years immediately preceding, and if the pension is to be the comfort it is meant to be to the respectable poor, it is hard that he should have to wait for his sixty-fifth birthday before he enjoys the little extra sum that represents his country's gratitude for his long laborious life.

